# Sublethal and Transgenerational Effects of Isocycloseram on the Life Table of Two-Spotted Spider Mites (*Tetranychus urticae*)

**DOI:** 10.3390/insects17060621

**Published:** 2026-06-12

**Authors:** Awad Ateia, Chunyan Yin, Zhiyuan Qin, Asanka Tennakoon, B. L. W. K. Balasooriya, Chao Shu, Zhenyu Wang

**Affiliations:** 1 State Key Laboratory of Green Pesticide, International Joint Research Center for Intelligent Biosensor Technology and Health, Central China Normal University, Wuhan 430079, China; 2 Department of Crop Protection, Faculty of Agriculture and Natural Resources, University of Bakht Alruda, Ed Dueim 28812, Sudan; 3 School of Life Sciences, Wuchang University of Technology, Wuhan 430223, China; 4 Department of Agricultural Technology, Faculty of Technology, University of Colombo, Pitipana, Homagama 10200, Sri Lanka; 5 Department of Biotechnology, Faculty of Agriculture and Plantation Management, Wayamba University of Sri Lanka, Makandura, Gonawila 60170, Sri Lanka

**Keywords:** *Tetranychus urticae*, isocycloseram, sublethal effect, life table, population-doubling time, integrated pest management

## Abstract

*Tetranychus urticae* is a highly destructive, polyphagous mite that has developed resistance to multiple acaricides, necessitating the evaluation of new compounds. Isocycloseram is a novel insecticide with potential to control this mite; the effects of its sublethal concentrations are still uninvestigated. In this study, an age-stage, two-sex life table model was used to evaluate the sublethal effects of isocycloseram concentrations (LC_10_ and LC_30_) on population growth, reproduction, and development of the two-spotted spider mite. The results showed that the LC_10_ and LC_30_ values were 0.012 mg/L and 0.022 mg/L, respectively. Sublethal concentrations of LC_10_ significantly affected population growth by reducing fertility, while LC_30_ significantly prolonged the immature stage and reduced average fecundity by 37%. With the LC_30_ treatment, the net reproductive rate *R*_0_ decreased by 43%, and the intrinsic rate of increase *r* decreased significantly, from 0.152 day^−1^ to 0.117 day^−1^. The doubling time *DT* was extended by 30%, from 4.55 days to 5.92 days. This study covers the importance of life table analysis for investigating sublethal effects and for ensuring that, when isocycloseram is incorporated into integrated pest management, both its direct toxicity and its effects on population dynamics are considered.

## Simple Summary

The two-spotted spider mite is a tiny pest that damages many food crops and has become resistant to numerous chemical controls. Scientists are therefore searching for new compounds that can effectively manage this pest. This study tested a novel insecticide, isocycloseram, against these mites. The experiments examined not only how many mites were killed by the chemical, but also how the survivors—and even their offspring—were affected. The results show that even very low, non-lethal doses of isocycloseram had strong negative effects. Mites exposed to these low doses took longer to develop from egg to adult and laid significantly fewer eggs. Consequently, the growth rate of the entire mite population slowed substantially, and the time required for the population to double increased by 30%. These findings are valuable because they demonstrate that isocycloseram can suppress mite populations well beyond simply killing the pests directly. Using such a compound in integrated pest management programs could help farmers protect their crops more sustainably while reducing the need for repeated spray applications.

## 1. Introduction

*Tetranychus urticae* Koch (Acari: Tetranychidae), commonly known as the two-spotted spider mite (TSSM), is a globally significant agricultural pest causing substantial economic damage to a wide range of crops [1,2]. The mite feeds on leaf parenchyma tissue, leading to chlorophyll loss and reduced photosynthetic capacity, which collectively result in considerable yield reductions in horticultural, ornamental, and agronomic production systems [3]. *T. urticae* exhibits extreme polyphagy, being documented on over 140 botanical families, including economically important vegetables such as beans, cucumbers, and peppers [4,5]. *T. urticae* is a small, highly polyphagous, and globally distributed invasive mite that exhibits a short developmental period and high reproductive rate, enabling rapid population increase under favorable conditions, especially in protected environments like greenhouses [6,7]. These biological attributes, combined with intensive acaricide use, have led to the widespread evolution of resistance against multiple chemical classes, including mitochondrial electron transport inhibitors (METI), pyrethroids, and organophosphates [2,8,9,10]. This resistance is usually driven by the enhanced activity of detoxifying enzymes such as cytochrome P450 and glutathione S-transferase [11]. To combat this persistent resistance, developing new compounds with novel mechanisms of action is paramount for sustainable pest management [12]. Isocycloseram is a newly developed isoxazoline insecticide that is as a powerful antagonist of gamma-aminobutyric acid (GABA) gated chloride channels [13], and is classified as Group 30 by the Insecticide Resistance Action Committee (IRAC) [14]. For this specific molecular target, no cross-resistance with other commonly used acaricide classes, such as METI inhibitors or pyrethroids, has been reported [15]. Furthermore, to date, no field resistance to IRAC Group 30 insecticides (including isocycloseram) has been documented in any insect or mite species, nor has cross-resistance with other GABAR-targeted insecticides, such as cyclodienes and fipronil, been reported [16,17]. Owing to its excellent bioactivity and low non-target toxicity, this compound has attracted widespread attention. Several studies have confirmed that isocycloseram is highly effective against Lepidoptera larvae, Coleoptera, thrips, public health pests, and spider mites (including *T. urticae*) [18,19,20,21,22]. As a result, isocycloseram provides a powerful new tool for controlling resistant populations of *T. urticae*.

Although determining the lethal effectiveness of new compounds is necessary, it is equally important to understand their sublethal effects. Sublethal effects are physiological and behavioral changes in surviving individuals, such as decreased fecundity, altered development, and feeding inhibition [23]. Most studies on isocycloseram have focused on lepidopteran pests, with no research on its sublethal effects on spider mite demographic traits. This knowledge gap is significant because sublethal doses can alter mite development, reproduction, and population growth across generations, ultimately determining the long-term success of isocycloseram in IPM. Therefore, the objectives of the present study were to: determine the LC_10_, LC_30_, and LC_50_ values of isocycloseram against *T. urticae* protonymphs; measure sublethal effects on the parental generation (fecundity, longevity, development); assess transgenerational effects on F_1_ offspring using age-stage, two-sex life table analysis; and project population-level impacts (net reproductive rate *R*_0_, intrinsic rate *r*, doubling time *DT*).

## 2. Materials and Methods

### 2.1. Tetranychus urticae Colonies and Acaricides

Individuals of *Tetranychus urticae* were collected from a field in Wuhan City, China. The mite colony was reared on potted *Phaseolus vulgaris* L. seedlings. Bean plants were grown in a greenhouse at 25 ± 2 °C, 60–70% relative humidity, with natural light supplemented by sodium lamps to maintain a 16:8 h (L:D) photoperiod. Mites were maintained in a climate-controlled chamber (State Key Laboratory of Green Pesticide, Central China Normal University) under the following conditions: temperature 26 ± 1 °C, relative humidity 60 ± 5%, and a photoperiod of 16:8 h (L:D). Fresh bean leaves were provided every 2–3 days to ensure adequate nutrition. The colony was maintained for one year without exposure to any acaricides before use in this study. Isocycloseram (purity > 98%) was obtained from Syngenta Group Co., Ltd. (Shanghai Pilot Free Trade Zone, Shanghai, China).

### 2.2. Bioassays for T. urticae

Bioassays were conducted on *Tetranychus urticae* protonymphs using the leaf-disc dipping method described by [24]. Protonymphs were collected from the laboratory colony over a 12–18 h period and were therefore not strictly age-synchronized; this reflects natural overlapping population structure, and the age-stage, two-sex life table analysis accounts for variable development rates [25].

Isocycloseram was first dissolved in dimethyl sulfoxide (DMSO) (purity ≥ 99%, Shanghai Macklin Biochemical Co., Ltd., Shanghai, China) to prepare a stock solution, then diluted with distilled water containing 0.1% Triton X-100 (Sinopharm Chemical Reagent Co., Ltd., Shanghai, China). The final DMSO concentration in all insecticide solutions was ≤0.01% (*v*/*v*). Preliminary range-finding tests determined the bioassay concentrations: a two-fold serial dilution of 0.5, 0.25, 0.125, 0.0625, 0.03125, and 0.015625 mg/L, plus an untreated control. The control solution consisted of distilled water with 0.1% Triton X-100. Bean leaf discs were cut to 3.5 cm in diameter. Each disc was infested with 10 protonymphs, dipped into the different concentrations for 5 s, briefly dried on filter paper, and left to air-dry at room temperature. The leaf discs were placed on moist, sterile absorbent cotton (Medical Grade, manufactured by Nanchang Enhui Medical Hygiene Materials Co., Ltd., Nanchang, China) inside sterile plastic Petri dishes (3.5 cm diameter, Beijing Lanjieke Technology Co., Ltd., Beijing, China). The Petri dish lids had a 1 cm diameter hole covered with a small cotton plug to allow air exchange while preventing mite escape. There were four replicates per concentration (10 mites per replicate, total 40 mites per concentration). After 24 h of exposure, protonymph mortality was recorded under a stereomicroscope, (Motic, Xiamen, China), and corrected mortality rates were calculated using Abbott’s formula.

### 2.3. Effect of Sublethal Acaricide Isocycloseram on Development, Reproduction, and Life Table Parameters of T. urticae

The sublethal effects of two concentrations (LC_10_, LC_30_) on *T. urticae* were studied using the leaf disc dipping method, with LC_10_ and LC_30_ values calculated from 24 h mortality bioassay data using probit analysis in Polo Plus. Fresh, intact bean leaves, each infested with approximately 60 protonymph mites, each representing an independent biological replicate, were carefully dipped for 5 s into solutions of LC_10_, LC_30_, or the control solution. Leaves were dried and placed on moist cotton in Petri dishes. After 24 h of exposure, surviving protonymphs from each treatment group were individually transferred to fresh, untreated bean leaf discs (3.5 cm diameter) placed on moist cotton. The surviving protonymph individuals per treatment served as the parental (P) generation for the life table study. Each parental (P) mite was monitored daily from the protonymph stage until death. Upon reaching adulthood, daily fecundity (egg count), adult female longevity, and adult male longevity were recorded. Leaf discs were replaced regularly to ensure food quality.

#### F_1_ Generation Development Study

Approximately 100 eggs per treatment were randomly collected from parental (P) cohorts during peak oviposition to establish F_1_ biological replicates. F_1_ eggs were monitored individually on fresh leaf discs. The duration of each developmental stage (egg, larva, protonymph, deutonymph) and survival were recorded daily. Upon adulthood, each newly emerged F_1_ female was paired with a male from the same treatment, and daily fecundity and longevity were recorded using the same protocol as for the parental generation.

### 2.4. Life Table Analysis

The raw data collected across the isocycloseram treatments (control, LC_10_, and LC_30_) were analyzed using the TWOSEXMSChart software (version as of 1 January 2025) [26]. The program implements the age-stage, two-sex life table theory described by [25], which is based on [27]. The age of each individual (in days) in each treatment was denoted by x, and the life stage by j.

Based on these, we calculated the development time and reproductive parameters. The equations for these parameters are given below, as described by [28,29]:

(1) $$s_{xj}=\frac{n_{xj}}{n_{0,1}}$$

where *S_xj_* is the age-stage-specific survival rate, *n_xj_* is the total number of individuals surviving to age *x* and stage *j*, and *n*_0,1_ is the total number of individuals used for the experiments.

(2) $$l_{x}=\sum_{j=1}^{m}S_{xj}$$

where *l_x_* is the age-specific survival rate, *m* is the number of stages, and *S_xj_* is the age-stage-specific survival rate.

(3) $$m_{x}=\frac{\sum_{j=1}^{m}S_{xj}f_{xj}}{\sum_{j=1}^{m}S_{xj}}$$

where *m_x_* is the age-specific fecundity of the population, *m* is the number of stages, *S_xj_* and *f_xj_* are the age-stage-specific survival rate and age-specific fecundity, respectively

Based on *S_xj_*, *l_x_*, and *m_x_*, the population growth parameters that determine the population growth rate, including the gross reproductive rate *GRR*, net reproductive rate *R*_0_, intrinsic rate of increase *r*, mean generation time *T*, and finite rate of increase *λ*, were calculated [27,30]. The following equations were used to calculate these parameters:

(4) $$GRR=\sum m_{x}$$

where *GRR* is the gross reproductive rate, *m_x_* is the age-specific fecundity of the population

(5) $$\sum_{x=0}^{\infty }l_{x}m_{x}=R_{0}$$

where *R*_0_ is the Net reproductive rate, *l_x_* is the age-specific survival rate, and *m_x_* is the age-specific fecundity of the population

(6) $$r=\sum_{x=0}^{\infty }e^{-r\left(x+1\right)}l_{x}m_{x}=1$$

where *r* is the Intrinsic rate of increase, *l_x_* is the age-specific survival rate, and *m_x_* is the age-specific fecundity of the population

(7) $$T=\frac{\ln R_{0}}{r}$$

where *T* is the mean generation time that represents the time from one generation to the next one

(8) $$\lambda =\sum_{n=1}^{\infty }\left(\lambda^{-\left(x+1\right)}\sum_{j=1}^{m}f_{xj}S_{xj}\right)=1$$

where *λ* is the finite rate of increase) m is the number of stages, *S_xj_* and *f_xj_* are the age-stage-specific survival rate and age-specific fecundity, respectively.

Furthermore, we computed the reproductive value *(v_xj_*), which is defined as the contribution of individuals of age x and stage j to the future population as follows:

(9) $$v_{xj}=\frac{e^{r\left(x+1\right)}}{S_{xj}}\sum_{i=x}^{\infty }e^{-r\left(i+1\right)}\sum_{y=j}^{m}S_{iy}^{\prime }f_{iy}$$

where v*_xj_* is the age-stage reproductive value

In addition, we calculated the Age-stage life expectancy *e_xj_*, which is defined as the time that an individual of age *x* and stage *j* is expected to live

(10) $$e_{xj}=\sum_{i=x}^{\infty }\sum_{y=j}^{m}S_{iy}^{\prime }$$

where $e_{xj}$ is the age-stage life expectancy, *S′_iy_* is the probability that an individual of age x and stage j will survive to age i and stage y by assuming *S_x_*_j_ = 1.

### 2.5. Statistical Analyses

The toxicity of isocycloseram to the protonymph was analyzed using POLO-Plus (Version 2.0, LeOra Software) [31]. This analysis estimated the lethal concentrations (LC_10_, LC_30_, and LC_50_), along with their corresponding 95% confidence intervals, and the slope of the concentration-mortality regression. Sublethal treatments on the developmental and reproductive parameters (e.g., fecundity (no. eggs), adult female longevity, and adult male longevity) measured in the parental generation (P) were examined using a one-way analysis of variance, and the mean differences were determined using Tukey’s HSD (*p* ≤ 0.05). The life table parameters of F_1_, such as the gross reproductive rate *GRR*, mean generation time *T*, net reproduction rate (*R*_0_), intrinsic rate of increase *r*, and finite rate of increase *λ*, were analyzed based on the theory of the age-stage two-sex life table [25,27], using the TWOSEX-MSchart program (version of 1 January 2025). Bootstrap analysis with 100,000 resamples was used to estimate the mean values and standard errors of various life-table and biological parameters [32,33]. Differences between development time and reproductive values were estimated using the paired bootstrap test in the TWOSEXMSChart (*p* < 0.05) [34,35]. To create the graphs of the demographic parameters, OriginPro 2025b (OriginLab Corp., Northampton, MA, USA) was used.

## 3. Results

### 3.1. Toxicity of Isocycloseram

The toxicity test for *Tetranychus urticae* protonymph determined the sublethal level of isocycloseram after 24 h (Table 1). The estimated LC_10_, LC_30,_ and LC_50_ values were 0.012, 0.022, and 0.034 mg/L, respectively.

### 3.2. Sublethal Effects of Isocycloseram on the Parental Generation (P)

The biological parameters of the parental generation of the Tetranychus urticae population exposed to the sublethal concentrations (LC_10_ and LC_30_) and control treatment are shown in Table 2.

The biological parameters of the parental generation were significantly affected by the sublethal concentrations of the isocycloseram. The fecundity of adult females that were exposed to sublethal concentrations of isocycloseram was significantly different. The LC_10_ and LC_30_ treatments reduced egg production by 28% and 37%, respectively, compared with the control. The longevity of female adults in the control treatment was higher than in the LC_10_ and LC_30_ treatments. However, there is no significant difference in female longevity between the LC_10_ and LC_30_. Male longevity shows a different pattern; there is no significant difference between the control and the LC_10_ treatment. However, the LC_30_ treatment causes a significant drop, reducing male longevity by 15% compared to the control.

### 3.3. Sublethal Effects of Isocycloseram on Biological Parameters of the F_1_ Generation

Exposure to sublethal concentrations of isocycloseram (LC_10_ and LC_30_) significantly altered the developmental parameters of *Tetranychus urticae* (Table 3).

Egg duration was significantly prolonged under LC_30_ (*p* < 0.00001) compared with the control and LC_10_, which were statistically similar. Larval duration: a clear, concentration-dependent increase was observed (Control: 1.41 d; LC_10_: 1.65 d; LC_30_: 1.89 d) (*p* < 0.00001). Protonymph Duration (d): The protonymph duration was significantly shorter under LC_30_ (1.86 d) than under the control (2.04 d) and LC_10_ (2.00 d) (*p* < 0.00041). Deutonymph Duration (d): No significant differences were found between LC_30_ and LC_10_ treatments (*p* < 0.0621), but there were significant differences between LC_30_ and control (*p* < 0.00775). Total Pre-adult Duration (d): The sum of all immature stages (egg to deutonymph). This critical fitness parameter showed a significant, stepwise increase with stress intensity (Control: 8.43 d; LC_10_: 8.81 d; LC_30_: 9.66 d) (*p* < 0.00001). Adult Pre-oviposition Period (APOP). The LC_10_ treatment caused a significant delay (3.75 d) (*p* < 0.02034) compared with the LC_30_ treatment, which resulted in the shortest APOP (3.18 d). Total Pre-oviposition Period (TPOP, d): TPOP was longest under LC_30_ (12.81 d), followed by LC_10_ (12.64 d), and shortest in the control (12.07 d), with significant differences between control and LC_30_ (*p* < 0.02187). Oviposition Days: The number of days during which a female actively lays eggs. This was significantly reduced in both sublethal treatments (Control: 11.03 d; LC_10_: 8.73 d; LC_30_: 9.66 d) (*p* < 0.00049), (*p* < 0.00696), respectively, with LC_10_ showing the most substantial reduction. Oviposition Period (d): The span between the first and last oviposition day, which can be longer than “oviposition days” if laying is intermittent. LC_10_ showed a significant reduction (*p* < 0.03759) compared with the control. Mean Fecundity (no. eggs/female): This is the most dramatically affected parameter, showing a sharp, concentration-dependent decline (Control: 35.78; LC_10_: 28.05; LC_30_: 23.19) (*p* < 0.00001). Sex-Specific Adult Longevity: Females, isocycloseram did not significantly alter lifespan (*p* < 0.14419). Male adults showed significant differences across control and LC_30_ only (*p* < 0.03862), whereas there were no significant differences across control and LC_10_.

### 3.4. Sublethal Effects of Isocycloseram on the Life Table of the F_1_

The demographic traits of the F_1_ generation showed reductions in the gross reproduction rate *GRR* (*p* = 0.01132), the net reproductive rate *R*_0_ (*p* = 0.00536), the intrinsic rate of increase *r* (*p* = 0.00346), the finite rate of increase *λ* (*p* = 0.00329), along with a significant prolongation of the doubling time *DT* (*p* = 0.0113). However, the mean generation time *T* was not significantly increased in LC_30_ (*p* = 0.1815) compared with the control (Table 4).

**Figure 1:**
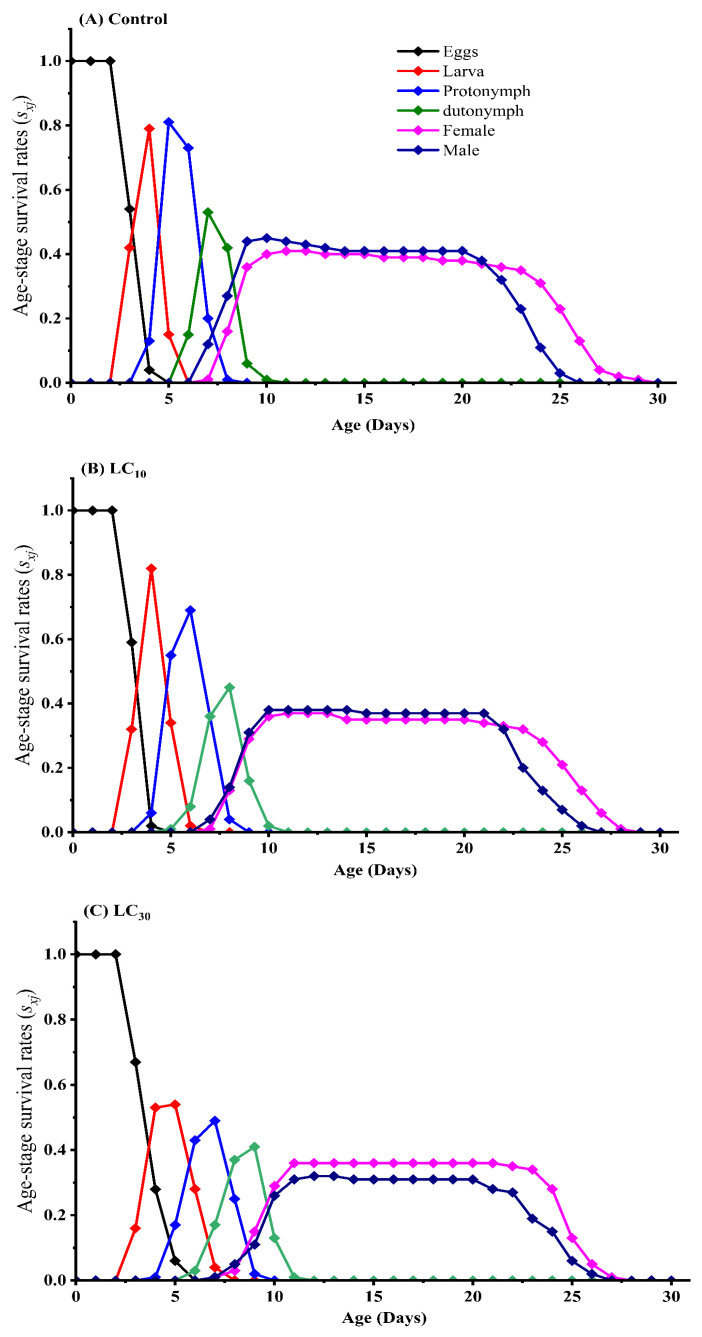
Age-stage specific survival rates (*s_x_*_j_) of the F_1_ offspring generation of *Tetranychus urticae*. Parents (P) were exposed to sublethal concentrations of Isocycloseram: (**A**) Control, (**B**) LC_10_, and (**C**) LC_30_.

Age-developmental-stage survival (*s_xj_*) curves in Figure 1. Total survival at age 10 days declined from 0.86 in the control to 0.76 and 0.68 under LC_10_ and LC_30_, respectively, and adult lifespan was shortened by approximately 2–4 days across treatments. Development was progressively delayed with increasing concentration: egg hatch was slower, immature stages (larva, protonymph, deutonymph) persisted longer, and peak adult emergence shifted from day 10–11 in the control to day 12–13 under LC_30_. The maximum proportion of adults reached 0.85 in the control, compared with 0.75 and 0.68 under LC_10_ and LC_30_, and adults disappeared 1–2 days earlier in treated cohorts (Figure 1B,C).

**Figure 2:**
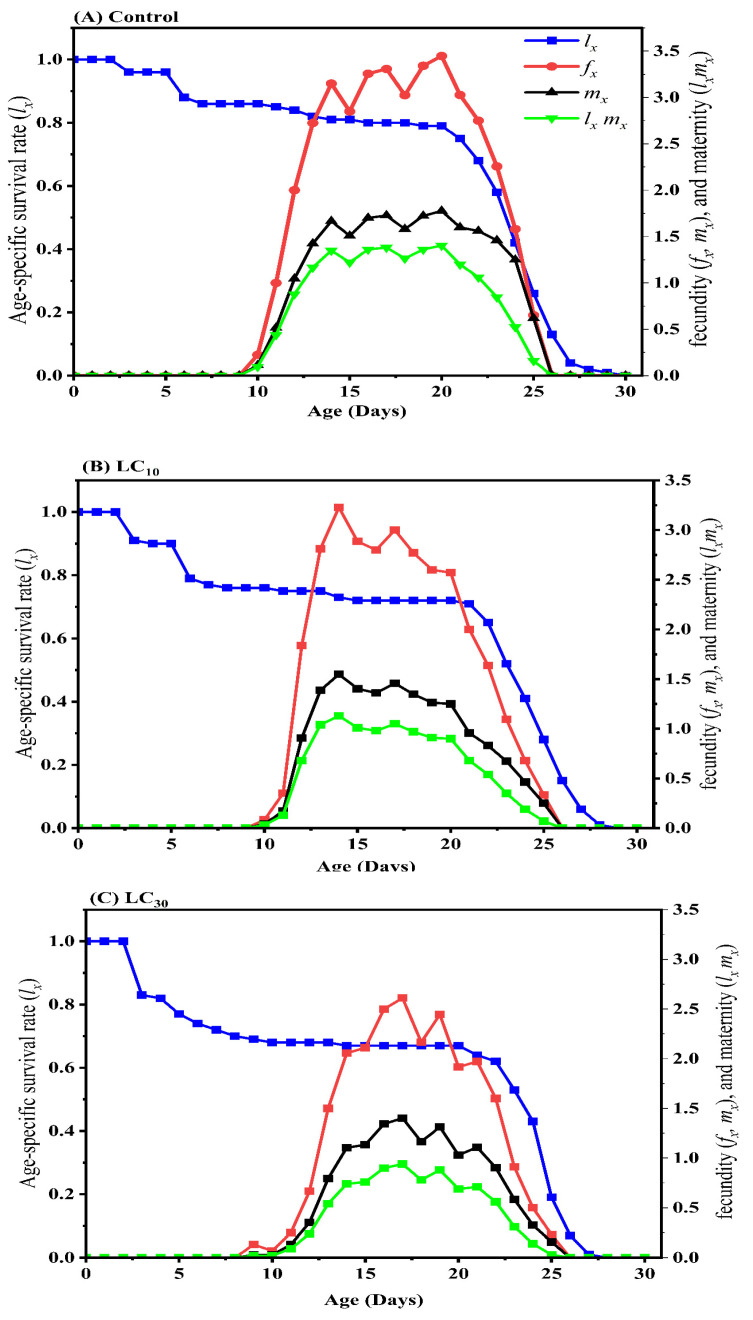
Age-specific survival rate (*l_x_*), age-specific fecundity (m_x_), and age-specific maternity (l_x_m_x_) of the F_1_ offspring generation of *Tetranychus urticae*. Parents (P) were exposed to sublethal concentrations of Isocycloseram: (**A**) Control, (**B**) LC_10_, and (**C**) LC_30_.

The age-specific life table data demonstrate that exposure to LC_10_ and LC_30_ significantly reduced both survival and reproductive output in a dose-dependent manner (Figure 2). In the control, age-specific survival (l_x_) remained above 0.8 until day 18 and declined gradually, whereas under LC_10_ and LC_30_, *l_x_* dropped more steeply, with values at day 18 of 0.72 and 0.67, respectively (Figure 2B,C). Female fecundity (*m_x_*) peaked at 1.66 eggs/female/day in the control, compared to 1.55 in LC_10_ and 1.40 in LC_30_, and the reproductive period was compressed under higher concentrations. The net reproductive rate *R*_0_, calculated as the sum of *l_x_ m_x_*, decreased from 14.67 in the control to 10.66 in LC_10_ and 8.35 in LC_30_, indicating that even sublethal concentrations substantially impair population replacement.

**Figure 3:**
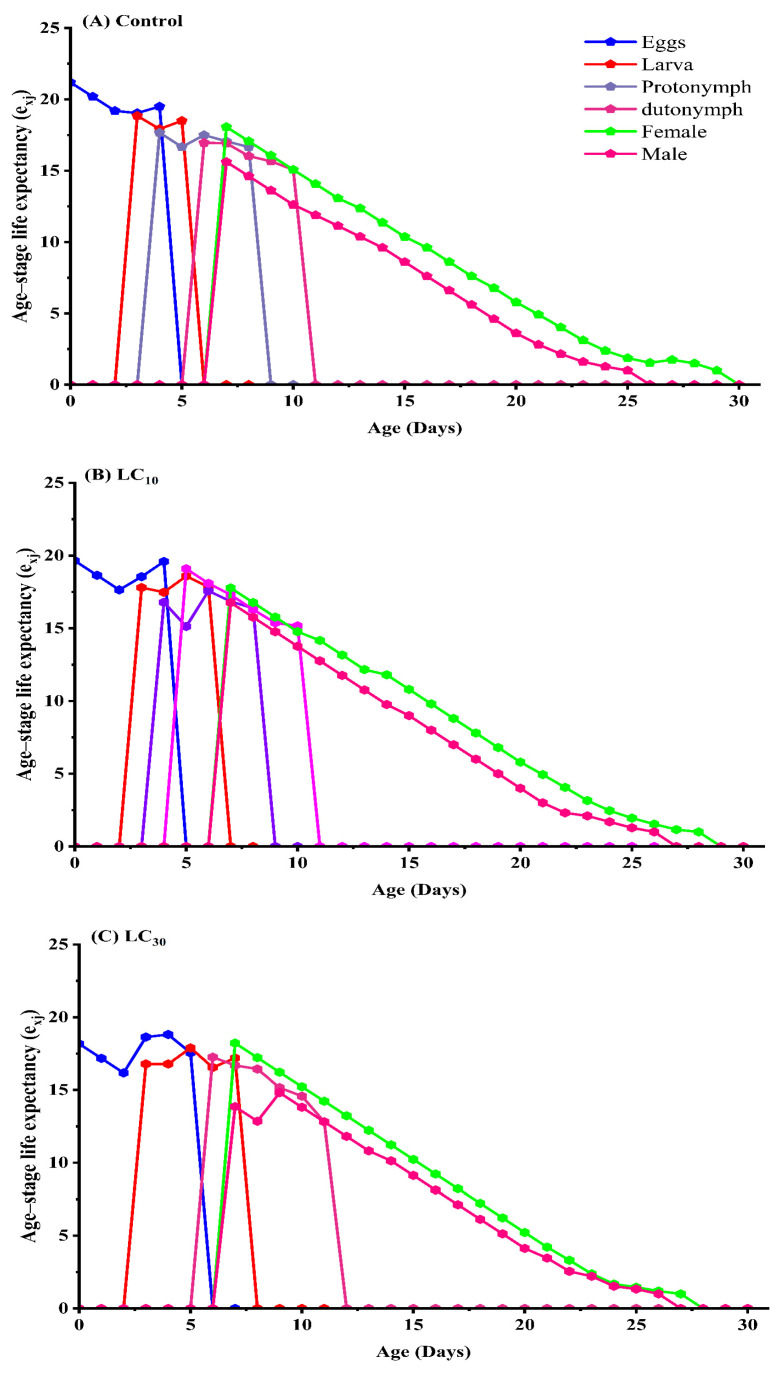
Age-stage specific life expectancy (*e_xj_*) for the F_1_ offspring of *Tetranychus urticae*. Parents (P) were exposed to sublethal concentrations of Isocycloseram: (**A**) Control, (**B**) LC_10_, and (**C**) LC_30_.

Exposure to LC_10_ and LC_30_ reduced age-stage life expectancy (*e_xj_*), in a concentration-dependent manner, particularly during early development. Life expectancy at birth (egg, age 0) declined from 21.2 days in the control to 19.6 days under LC_10_ and 18.2 days under LC_30_, indicating a substantial shortening of total lifespan (Figure 3). Throughout the immature stages (larva, protonymph, deutonymph), remaining life expectancy was consistently lower in treated cohorts, with the greatest reductions observed under LC_30_. For adult females and males that successfully emerged, life expectancy was often slightly higher under LC_10_ than in the control (e.g., female at age 10: 15.2 vs. 15.1 days) (Figure 3A,B).

**Figure 4:**
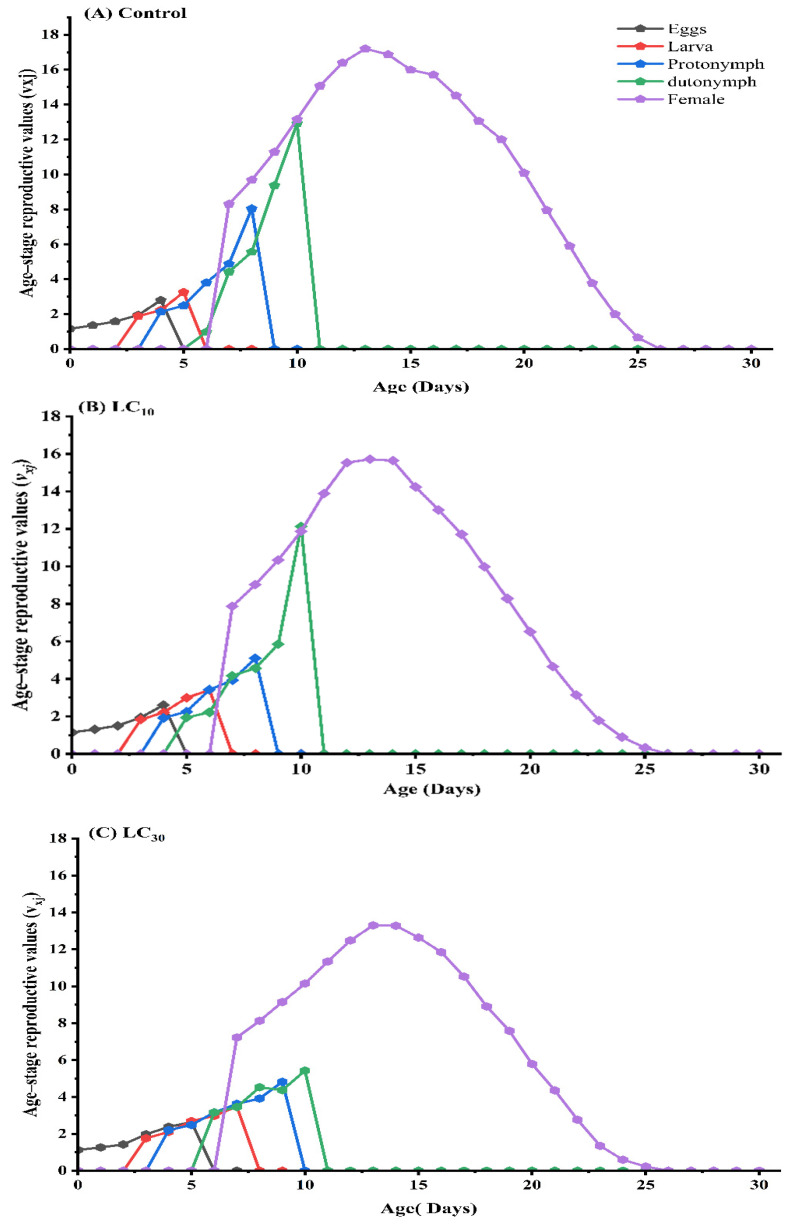
Age-stage reproductive values (*v_x_*_j_) of the F_1_ offspring generation of *Tetranychus urticae*. Parents (P) were exposed to sublethal concentrations of Isocycloseram: (**A**) Control, (**B**) LC_10_, and (**C**) LC_30_.

The age-stage reproductive value (*v_xj_*), which indicates the contribution of each individual at age x and stage j to the new population, is shown in Figure 4. Reproductive value at birth (egg, age 0) declined from 1.17 in the control to 1.14 in LC_10_ and 1.12 in LC_30_. For adult females, the peak reproductive value was significantly lower under treatment, reaching 17.20 at age 13 in the control, compared to 15.71 at age 13 in LC_10_ and 13.31 at age 13 in LC_30_, and declined more steeply thereafter. Immature stages (larvae, protonymphs, and deutonymphs) also exhibited consistently lower reproductive values under both concentrations.

**Figure 5:**
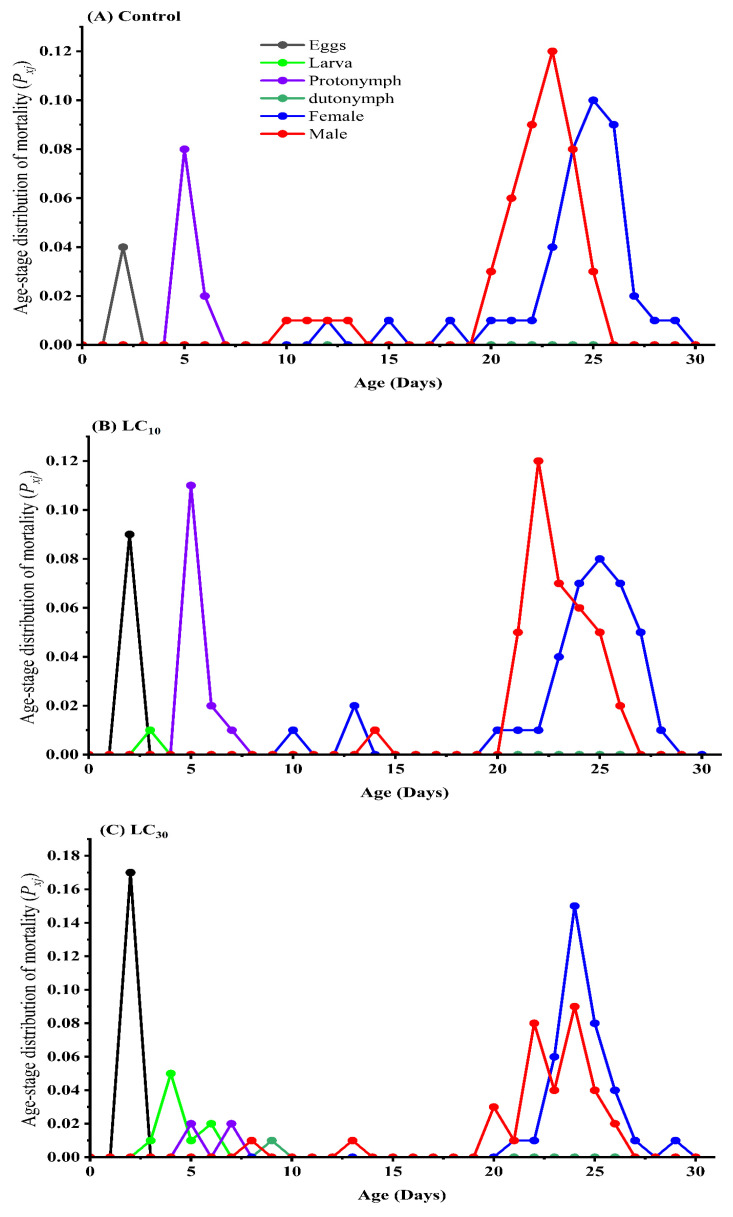
Age-stage distribution of mortality of the F_1_ offspring generation of *Tetranychus urticae*. Parents (P) were exposed to sublethal concentrations of Isocycloseram: (**A**) Control, (**B**) LC_10_, and (**C**) LC_30_.

Age-stage distribution of mortality (*P_xj_*) showed increased magnitude and breadth of death risks across the life cycle. In the control, mortality was concentrated in late adult life (days 20–29), with minor losses during the egg (2.5%), larval (8%), and protonymph (2%) stages (Figure 5). Under LC_10_, egg mortality rose to 9%, larval mortality appeared at age 3, and protonymph mortality increased to 11% at age 5, while adult female mortality was slightly elevated after day 20 (Figure 5B). LC_30_ intensified these effects: egg mortality reached 17%, larval mortality occurred repeatedly (ages 3–6), and deutonymphs experienced 1% mortality at age 9, a stage virtually unaffected in controls (Figure 5C). Adult female mortality under LC_30_ peaked sharply at age 24 (15%), exceeding control levels, and male mortality was also redistributed (Figure 5C).

## 4. Discussion

Pesticides have both direct and indirect effects on pests via sublethal impacts, that is, effects that are not lethal but still influence pests’ growth, development, and reproductive capacity. According to [36], sublethal effects can also influence all generations of a species (including offspring), and responses can vary among individuals [37,38]. It is important to find a pesticide that is effective against the pest and safe or less harmful to its natural enemies [39]. This study provides population parameters and demographic data on offspring in treated *T. urticae*. The demographic response of *T. urticae* to sublethal isocycloseram is complex. However, it follows life history theory, where organisms trade off resources among growth, development, maintenance, and reproduction under stress [40,41]. In this study, the results indicated that LC_10_ and LC_30_ possess high costs, particularly through reduced fecundity, development, and reproduction. The primary response was to prolong the pre-adult development period at increasing sublethal concentrations, a classic compensatory survival mechanism that enables the insect to metabolize the insecticides. The significant increase in pre-adult duration from 8.43 days in the control to 9.66 days at LC_30_ (*p* < 0.05) suggests that individuals spent more time on somatic maintenance and detoxification at the expense of developmental speed. This increase in duration is especially evident during egg hatching and larval stages; this pattern has been documented to increase under sublethal pesticide exposure in the two-spotted spider mite, *T. urticae* [37,42]. While this extension can be viewed as a survival strategy that facilitates toxin metabolism, it entails ecological costs, including prolonged exposure to natural enemies and delayed reproduction, which can affect population growth rates.

The deepest impact of sublethal effects is on reproductive parameters, revealing a clear survival-reproduction trade-off. Female adult longevity was unaffected among treatments, while male adult longevity was slightly affected among LC_30_ compared with the control. A similar result was observed in some resistant strains or under stress from pesticides, where survival mechanisms are prioritized over reproduction [43]. Accordingly, all parameters of reproductive decline; mean fecundity dropped by 22% at LC_10_ and 35% at LC_30_, with a significant reduction in oviposition days and periods. In this regard, numerous studies have also indicated that exposure to sublethal concentrations of acaricides can reduce the fecundity and oviposition days of treated *T. urticae* compared to controls [44,45,46], and they have reported similar results in *T. urticae* with diflovidazin, chlorfenapyr, and fluralaner. Such an effect on reproductive parameters indicates a redirection of energy resources from reproduction to the preservation of physiological functions and stress tolerance. A decrease in the pre-oviposition period in adult insects at the concentration of LC_30_ compared to the concentration of LC_10_ may indicate a greater effort to reproduce quickly before death occurs.

An unexpected finding was the significantly shorter protonymph duration under LC_30_ (1.86 d) compared with the control (2.04 d). While most stressors prolong development, this accelerated development may reflect a hormetic response—a low-dose stimulation that allows mites to reach reproductive age faster under chemical stress. For instance, sublethal diflovidazin significantly reduced female maturation duration in *T. urticae* [45], and similarly, sublethal avermectin shortened the larval and nymphal developmental periods of the citrus red mite *Panonychus citri* [47]. Alternatively, accelerated development could represent a compensatory mechanism to offset other fitness costs. The phenomenon is consistent with insecticide-induced hormesis, which has been linked to pest resurgence and altered population dynamics [48].

For the demographic trait, translating these individual-level effects into population growth parameters in the F_1_ generation (Table 4) confirms the significant demographic impact of parental exposure. The most important of these parameters is the gross reproductive rate *GRR* and, crucially, the net reproductive rate *R*_0_. *R*_0_ declined by approximately 43% at LC_30_, indicating a severe reduction in the population’s replacement rate. This decline directly stems from the observed decrease in mean fecundity. As a result, the intrinsic rate of increase *r*, decreased significantly from 0.152 to 0.117 day^−1^ in LC_30_. This aligns with previous studies showing that sublethal effects of spirodiclofen, abamectin, and pyridaben significantly reduce *R*_0_ and *r* in *T. urticae* [49]. Although the finite rate of increase *λ* decreased significantly only at LC_30_ compared to control, while LC_10_ was intermediate and not significantly different, its value near 1.0 indicates a change from rapid growth toward population stability. Doubling Time *DT* increased from 4.55 days in the control to 5.92 days under LC_30_, a 30% increase; this result aligns with studies reported by [45]. A longer *DT* showed that a spider mite population exposed to sublethal isocycloseram would require substantially more time to recover from sublethal exposure and then reach damaging thresholds, offering a critical point for integrated pest management (IPM) interventions. Prolongation of *DT* is a beneficial sublethal effect from a management perspective and has been observed with other compounds that reduce fecundity and the intrinsic rate of increase *r*. In demographic theory, mean generation time *T* is sensitive to changes in the age of first reproduction, but in this study, a paradox emerges between the prolonged pre-adult duration (TPOP) and the stability of the mean generation time *T* across treatments. Its stability suggests that, although juvenile development was delayed, other compensatory factors, such as the aforementioned shift in APOP or subtle changes in age-specific fecundity, moderated its effect in the integrated model. This documented the complexity of life-history responses and the critical importance of using comprehensive two-sex life-table analyses rather than single-sex life-table parameter assessments. The observed demographic suppression can be mechanistically due to the mode of action of isocycloseram as a non-competitive antagonist of GABA-gated chloride channels. Sublethal neuronal disruption likely imposes physiological costs, diverting energy from reproduction, reducing *GRR* and *R*_0_, and potentially altering hormonal regulation of development. Transgenerational consequences of sublethal pesticide exposure have been documented in other insect systems, where sublethal concentrations lead to fitness-related phenotypic changes that extend across generations [50]. Non-genetic inheritance mechanisms, including egg provisioning, epigenetic modifications, and altered gene expression, may mediate these effects. In line with this pattern, the significant effects observed in the F_1_ generation of *T. urticae* provide empirical support for either across-generations epigenetic modifications or reduced maternal provisioning under isocycloseram stress.

## 5. Conclusions

This study on sublethal concentrations showed that isocycloseram, particularly at the LC_30_ level, induces significant life-history changes in Tetranychus urticae that profoundly suppress population growth potential in both the parental (P) and F_1_ generations. The primary results demonstrated that isocycloseram exerts its demographic impact by extending the pre-adult stage and reducing fecundity. These individual-level disruptions culminate in a 43% decline in the net reproductive rate *R*_0_ and a 30% increase in the population doubling time *DT*, indicating a significantly limited capacity for population recovery. However, future studies are still needed to investigate isocycloseram-induced sublethal effects across multiple generations at the biological and molecular levels and to elucidate the underlying mechanisms of pesticide resistance.

## Figures and Tables

**Table 1:** Lethal concentration of Isocycloseram on *Tetranychus urticae* protonymph in laboratory bioassays 24 h after exposure.

Acaricide	N	LC_10_(95%CI) mg/L	LC_30_(95%CI) mg/L	LC_50_(95%CI) mg/L	*χ*^2^ (df)	Slope ± SE	*p*-Value
Isocycloseram	280	0.012 (0.008–0.016)	0.022 (0.017–0.028)	0.034 (0.027–0.041)	0.536 (4)	2.854 ± 0.371	0.901

Note: 95%, CI: Confidence Interval; SE, Standard error; χ^2^, Chi square value; df, Degrees of freedom.

**Table 2:** Sublethal effects of isocycloseram on life table parameters of *Tetranychus urticae* in parental generation.

Parameters	Control	LC_10_	LC_30_
Fecundity (no. eggs)	36.083 ± 0.692 a (24)	25.875 ± 0.618 b (22)	22.875 ± 0.406 c (19)
Adult female longevity (days)	17.875 ± 0.819 a (24)	16.083 ± 0.509 b (22)	16.125 ± 0.341 b (19)
Adult male longevity (days)	12.667 ± 0.628 a (20)	12.500 ± 0.296 a (20)	10.708 ± 0.236 b (16)

LC_10_ and LC_30_ refer to the lethal concentrations causing 10% and 30% mortality, respectively. Values are presented as mean ± SE. Different lowercase letters (a, b, c) within the same row indicate significant differences among treatments (control, LC_10_, LC_30_) as determined by one-way ANOVA followed by Tukey’s HSD test (*p* < 0.05). The same letter indicates no significant difference. Values in parentheses indicate the number of individuals evaluated.

**Table 3:** Effects of sublethal isocycloseram on life table parameters of *Tetranychus urticae* in the F_1_ generation.

Parameters	Control	LC_10_	LC_30_
Egg (♀ + ♂) (days)	3.60 ± 0.058 b (96)	3.67 ± 0.054 b (91)	4.21 ± 0.092 a (83)
Larva (♀ + ♂) (days)	1.41 ± 0.053 c (96)	1.65 ± 0.053 b (90)	1.89 ± 0.060 a (74)
Protonymph (♀ + ♂) (days)	2.04 ± 0.033 a (86)	2.00 ± 0.046 a (76)	1.86 ± 0.042 b (70)
Deutonymph (♀ + ♂) (days)	1.36 ± 0.057 b (86)	1.42 ± 0.066 a (76)	1.59 ± 0.06 a (69)
Pre-adult (♀ + ♂) (days)	8.43 ± 0.093 c (86)	8.81 ± 0.103 b (76)	9.66 ± 0.120 a (69)
APOP (days)	3.32 ± 0.22 ab (40)	3.75 ± 0.23 a (37)	3.18 ± 0.11 b (36)
TPOP (days)	12.07 ± 0.27 b (40)	12.64 ± 0.231 ab (37)	12.81 ± 0.181 a (36)
Oviposition days	11.025 ± 0.48 a (40)	8.73 ± 0.44 c (37)	9.66 ± 0.28 b (36)
Oviposition period (days)	11.30 ± 0.61 a (40)	9.64 ± 0.69 b (37)	10.41 ± 0.31 a (36)
Mean fecundity (no. eggs)	35.78 ± 1.24 a (41)	28.05 ± 1.68 b (38)	23.19 ± 0.98 c (37)
Female adult longevity (days)	16.34 ± 0.504 a (41)	15.87 ± 0.67 a (38)	15.55 ± 0.190 a (37)
Male adult longevity (days)	14.89 ± 0.523 a (38)	13.69 ± 0.56 ab (32)	13.49 ± 0.56 b (31)

Note: Values are means ± SE. Different lowercase letters (a, b, c) within the same row indicate significant differences among treatments (control, LC_10_, LC_30_) as determined by the paired bootstrap test (100,000 resamples; *p* < 0.05); APOP: Adult pre-oviposition period (time between adult emergence and first oviposition); TPOP: Total Pre-Ovipositional Period (duration from egg to first oviposition); Means were separated with a paired bootstrap test (*p* < 0.05) and standard errors by bootstrap with 100,000 samples. Values in parentheses indicate the number of individuals evaluated.

**Table 4:** Effects of sublethal isocycloseram on population parameters of *Tetranychus urticae* in F_1_ generation.

Parameters	Control	LC_10_	LC_30_
*GRR* (offspring)	19.85 ± 2.25 a (40)	15.31 ± 1.91 a (37)	12.91 ± 1.51 b (36)
*R*_0_ (offspring)	14.67 ± 1.920 a (40)	10.66 ± 1.500 a (37)	8.35 ± 1.150 b (36)
*r* (day^−1^)	0.152 ± 0.0081 a (40)	0.136 ± 0.0087 ab (37)	0.117 ± 0.0082 b (36)
***λ*** (day^−1^)	1.16 ± 0.0094 a (40)	1.14 ± 0.0099 ab (37)	1.12 ± 0.0092 b (36)
*T* (day)	17.54 ± 0.200 a (40)	17.27 ± 0.720 a (37)	17.94 ± 0.220 a (36)
*DT*	4.55 ± 0.240 b (40)	5.10 ± 0.330 a (37)	5.92 ± 0.430 a (36)
Relative fitness *R_f_*	-	0.72	0.57

Notes: Values are presented as mean ± SE. Different lowercase letters (a, b) within the same row indicate significant differences among treatments (control, LC_10_, LC_30_) as determined by the paired bootstrap test (100,000 resamples; *p* < 0.05). *GRR*, gross reproductive rate; *R*_0_, net reproductive rate; *r*, intrinsic rate of increase; ***λ***, finite rate of increase; *DT*, doubling time; *T*, mean generation time. Relative fitness = *R*_0_ (LC_10_ and LC_30_)/*R*_0_ (Control). Values in parentheses indicate the number of individuals evaluated.

## Data Availability

The original contributions presented in this study are included in the article/Supplementary Materials. Further inquiries can be directed to the corresponding author.

## Supplementary Materials

The following supporting information can be downloaded at: https://www.mdpi.com/article/10.3390/insects17060621/s1, Figure S1: Concentration-response curve of isocycloseram against *Tetranychus urticae* protonymphs after 24 h exposure (pooled data from three replicates); Table S1: Probit analysis of isocycloseram toxicity against *Tetranychus urticae* protonymphs after 24 h exposure—three replicate bioassays; Dataset S2: Raw life table data (individual stage durations, sex, and longevity) for control, LC_10_, and LC_30_ treatments of *Tetranychus urticae* used in the age-stage, two-sex life table analysis; Supplementary File S3: Bootstrap output (100,000 resamples) from TWOSEX-MSChart for life table parameters of *Tetranychus urticae* under control, LC_10_, and LC_30_ treatments.
