# Supplementary material for: Sublethal and Transgenerational Effects of Isocycloseram on the Life Table of Two-Spotted Spider Mites (Tetranychus urticae)
Source: Insects. 2026 Jun 12;17(6):621. doi: 10.3390/insects17060621 (PMC13299980; doi:10.3390/insects17060621)
Supplement: Supplementary file 1 [file insects-17-00621-s001.zip › Bootstrap_Outputs_Control_LC10_LC30_S3.pdf]

Bootstrap output from TWOSEX-MSChart (100,000 resamples) for *Tetranychus urticae* life table parameters under control, LC_{10}, and LC_{30} treatments

- ### CONTROL (CK) Treatment

Program: Age-Stage, Two-Sex Life Table Analysis (TWOSEX-MSChart).
Table 40. Table of Bootstrap results
Project: Life table raw data. Mites. Ver. 9/26/2025
Username: AWAD. Pt:1W6P
Treatment: TSSM
Analyzed on: 10-14-2025
Bootstrap began: 14/10/2025 14:44:36 . Ended: 14/10/2025 14:46:53 . Total time: 2.28 min.
Bootstrap sample size: 100
Total number of bootstrap: 100000
Two-sex mating: Yes. Same bootstrap: No
For B = 1 million version, you will see "No data" in cases of no bootstrap results are estimated.

Table of bootstrapping results of population parameters (TWOSEX mating: Yes)
==

	r	lambda	R0	T	GRR	F	Nf/N	Longevity
ORIGINAL	0.153151	1.165501	14.67	17.537	19.85	35.78	0.41	21.2
Original n	100	100	100	100	100	41	100	100
B	100000	100000	100000	100000	100000	100000	100000	100000
Boot. max	0.181785	1.199356	23.24	18.597	30.24	43.14	0.64	23.79
Boot. mean	0.152676	1.16498	14.677	17.542	19.86	35.78	0.4102	21.2
Boot. min	0.104581	1.110245	6.72	16.732	10.95	25.68	0.2	17.74
Variance	0.00006	0.00009	3.72133	0.042	5.06	3.94	0.0024	0.52
S.E.	0.00816	0.009495	1.92908	0.205	2.25	1.984	0.0489	0.72

*** Use the "ORIGINAL" (parameter for the cohort) and SE in your paper. ***
*** For normal distribution and B>=100,000, the bootstrap mean will be close to ORIGINAL. ***
*** If it is a normal distribution, the percentile confidence intervals (PCI) will be very ***
*** close to the confidence intervals calculated using the t-value (TCI). ***
*** If original-SE or original+SE gives illogical results (e.g., R0-SE < 0, F-SE < 0, ***
*** lambda-SE < 0, etc.), you should report the PCI, not SE. ***
****

*** The longevity is the total longevity (from birth to death). ***
*** Because GRR ignores the survival rate, it is not a good statistic. ***
==

Attention!
SE of intrinsic rate according to Efron: 8.15952809457009E-03 (Double check passed).
Original are the parameters calculated using all individuals.
r: intrinsic rate, lambda: finite rate, R0: net reproduction rate,
T: mean generation time, GRR: gross reproduction rate.
F: mean fecundity per female adult.
Nf/N: it is the proportion of female adults in total individuals.
According to Chi (1988), Ro=F*(Female%), this result is consistent with Chi (1988).
Effective bootstraps are bootstraps with non-zero net reproductive rate.

LC_{10} Treatment

Program: Age-Stage, Two-Sex Life Table Analysis (TWOSEX-MSChart).
Table 40. Table of Bootstrap results
Project: Life table raw data. Mites. Ver. 9/26/2025
Username: awad. Pt:3r5d
Treatment: TSSM
Analyzed on: 10-14-2025
Bootstrap began: 14/10/2025 21:46:08 . Ended: 14/10/2025 21:48:22 . Total time: 2.23 min.
Bootstrap sample size: 100
Total number of bootstrap: 100000
Two-sex mating: Yes. Same bootstrap: No
For B = 1 million version, you will see "No data" in cases of no bootstrap results are estimated.

Table of bootstrapping results of population parameters (TWOSEX mating: Yes)
==

	r	lambda	R0	T	GRR	F	Nf/N	Longevity
ORIGINAL	0.137016	1.146847	10.66	17.272	15.31	28.05	0.38	19.64
Original n	100	100	100	100	100	38	100	100
B	100000	100000	100000	100000	100000	100000	100000	100000
Boot. max	0.168032	1.182974	17.79	18.372	25.22	35.61	0.61	23.25
Boot. mean	0.136431	1.146219	10.66176	17.277	15.31	28.05	0.3801	19.64
Boot. min	0.093776	1.098314	4.98	16.422	7.27	20.21	0.19	14.99
Variance	0.000076	0.0001	2.26634	0.047	3.65	2.83	0.0024	0.76
S.E.	0.00873	0.009997	1.50544	0.217	1.911	1.681	0.0487	0.871

*** Use the "ORIGINAL" (parameter for the cohort) and SE in your paper. ***
*** For normal distribution and B>=100,000, the bootstrap mean will be close to ORIGINAL. ***
*** If it is a normal distribution, the percentile confidence intervals (PCI) will be very ***
*** close to the confidence intervals calculated using the t-value (TCI). ***
*** If original-SE or original+SE gives illogical results (e.g., R0-SE < 0, F-SE < 0, ***
*** lambda-SE < 0, etc.), you should report the PCI, not SE. ***
****
*** The longevity is the total longevity (from birth to death). ***
*** Because GRR ignores the survival rate, it is not a good statistic. ***
==
Attention!
SE of intrinsic rate according to Efron: 8.73347333442352E-03 (Double check passed).
Original are the parameters calculated using all individuals.
r: intrinsic rate, lambda: finite rate, R0: net reproduction rate,
T: mean generation time, GRR: gross reproduction rate.
F: mean fecundity per female adult.
Nf/N: it is the proportion of female adults in total individuals.
According to Chi (1988), Ro=F*(Female%), this result is consistent with Chi (1988).
Effective bootstraps are bootstraps with non-zero net reproductive rate.

LC$_{30}$ Treatment

- Program: Age-Stage, Two-Sex Life Table Analysis (TWOSEX-MSChart).

Table 40. Table of Bootstrap results
Project: Life table raw data. Mites. Ver. 9/26/2025
Username: AWAD. Pt:9c7e
Treatment: TSSM
Analyzed on: 10-15-2025
Bootstrap began: 15/10/2025 15:33:47 . Ended: 15/10/2025 15:36:15 . Total time: 2.47 min.
Bootstrap sample size: 100
Total number of bootstrap: 100000
Two-sex mating: Yes. Same bootstrap: No
For B = 1 million version, you will see "No data" in cases of no bootstrap results are estimated.

Table of bootstrapping results of population parameters (TWOSEX mating: Yes)

==

	r	lambda	R0	T	GRR	F	Nf/N	Longevity
ORIGINAL	0.118238	1.125512	8.35	17.949	12.91	23.19	0.36	18.17
Original n	100	100	100	100	100	36	100	100
B	100000	100000	100000	100000	100000	100000	100000	100000
Boot. max	0.145604	1.156738	13.29	18.926	19.12	26.6	0.57	22.08
Boot. mean	0.117675	1.124916	8.34533	17.953	12.91	23.19	0.3598	18.17
Boot. min	0.069313	1.071771	3.52	16.615	5.97	19.21	0.16	13.92
Variance	0.000067	0.000085	1.32388	0.053	2.31	0.61	0.0023	0.91
S.E.	0.00821	0.009221	1.1506	0.23	1.52	0.782	0.0481	0.952

*** Use the "ORIGINAL" (parameter for the cohort) and SE in your paper. ***
*** For normal distribution and B>=100,000, the bootstrap mean will be close to ORIGINAL. ***
*** If it is a normal distribution, the percentile confidence intervals (PCI) will be very ***
*** close to the confidence intervals calculated using the t-value (TCI). ***
*** If original-SE or original+SE gives illogical results (e.g., R0-SE < 0, F-SE < 0, ***
*** lambda-SE < 0, etc.), you should report the PCI, not SE. ***
****
*** The longevity is the total longevity (from birth to death). ***
*** Because GRR ignores the survival rate, it is not a good statistic. ***

==

Attention!
SE of intrinsic rate according to Efron: 8.20757022715353E-03 (Double check passed).
Original are the parameters calculated using all individuals.
r: intrinsic rate, lambda: finite rate, R0: net reproduction rate,
T: mean generation time, GRR: gross reproduction rate.
F: mean fecundity per female adult.
Nf/N: it is the proportion of female adults in total individuals.
According to Chi (1988), Ro=F*(Female%), this result is consistent with Chi (1988).
Effective bootstraps are bootstraps with non-zero net reproductive rate.

References to cite:

Efron, B. and Tibshirani, R. J. 1993. An Introduction to the Bootstrap. Chapman & Hall, New York, USA.
Huang, Y. B. and Chi, H. 2013. Life tables of Bactrocera cucurbitae (Diptera: Tephritidae): with an invalidation of the jackknife technique Journal of Applied Entomology 137: 327-339. (You should cite this paper)

****
**
