# Supplementary material for: Sublethal and Transgenerational Effects of Isocycloseram on the Life Table of Two-Spotted Spider Mites (Tetranychus urticae)
Source: Insects. 2026 Jun 12;17(6):621. doi: 10.3390/insects17060621 (PMC13299980; doi:10.3390/insects17060621)
Supplement: Supplementary file 1 [file insects-17-00621-s001.zip › Table S1.pdf]

Table S1. Probit analysis of isocycloseram toxicity against *Tetranychus urticae* protonymph after 24 h exposure – three replicate bioassays.

Replicate	N	Slope ± SE	χ^2 (df)	P-value	LC_{10} (mg/L) (95% CI)	LC_{30} (mg/L) (95% CI)	LC_{50} (mg/L) (95% CI)
1 (iso1)	280	2.854 ± 0.371	0.536 (4)	0.901	0.012 (0.008–0.016)	0.022 (0.017–0.028)	0.034 (0.027–0.041)
2 (iso11)	280	3.101 ± 0.401	0.365 (4)		0.014 (0.009–0.018)	0.024 (0.018–0.029)	0.035 (0.029–0.042)
3 (iso111)	280	2.996 ± 0.399	0.801 (4)		0.014 (0.009–0.018)	0.025 (0.018–0.030)	0.037 (0.030–0.044)
