# Supplementary figures and images for: Sublethal and Transgenerational Effects of Isocycloseram on the Life Table of Two-Spotted Spider Mites (Tetranychus urticae)
Source: Insects. 2026 Jun 12;17(6):621. doi: 10.3390/insects17060621 (PMC13299980; doi:10.3390/insects17060621)

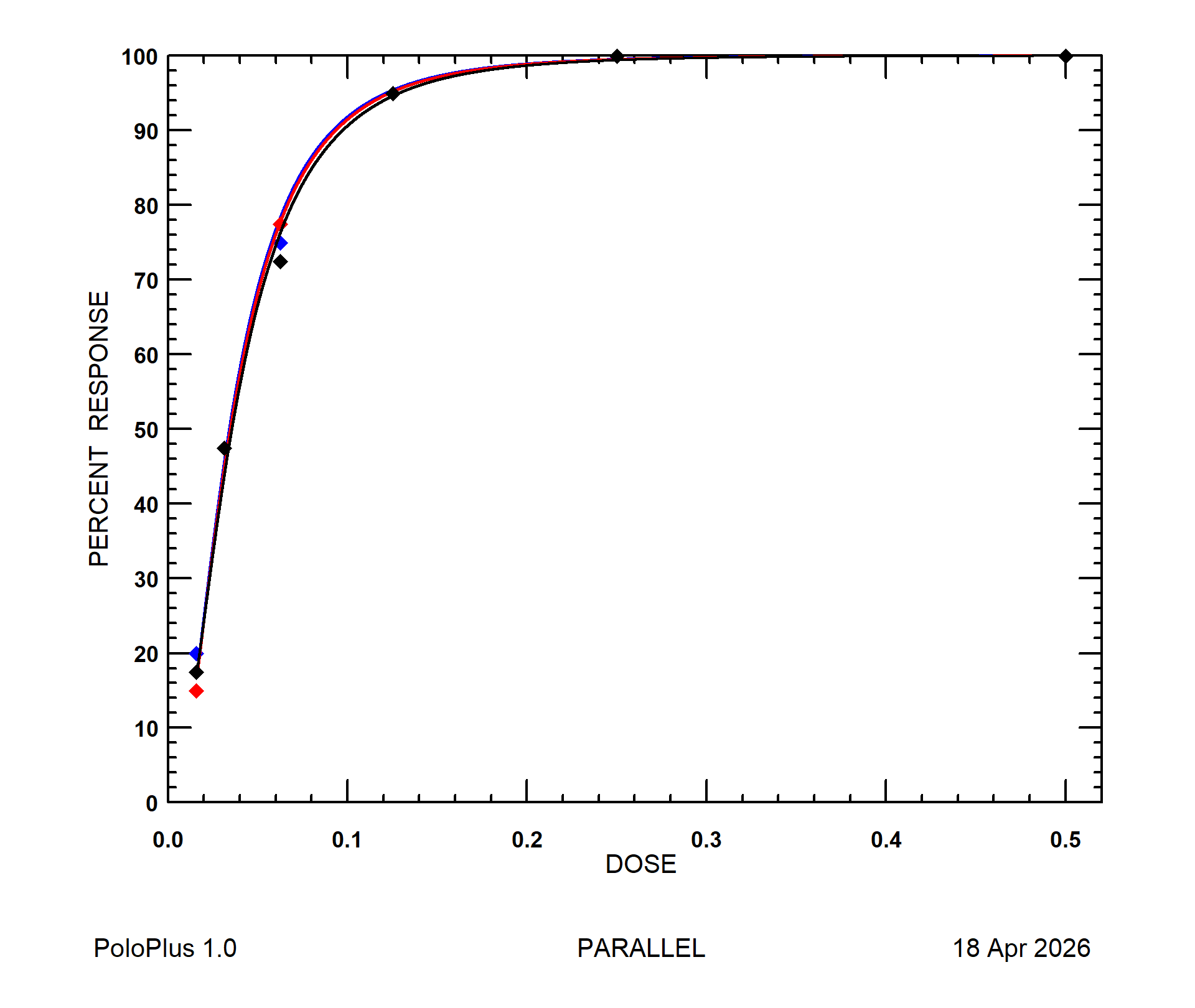

Supplement: Supplementary file 1 [file insects-17-00621-s001.zip › Figure S1.BMP]
